# QTL mapping and genomic prediction of resistance to wheat head blight caused by *Fusarium verticillioides*

**DOI:** 10.3389/fgene.2022.1039841

**Published:** 2022-10-24

**Authors:** Junqiao Song, Yuhui Pang, Chunping Wang, Xuecai Zhang, Zhankui Zeng, Dehui Zhao, Leiyi Zhang, Yong Zhang

**Affiliations:** ^1^ College of Agronomy, Henan University of Science and Technology, Luoyang, China; ^2^ The Shennong Laboratory, Zhengzhou, Henan, China; ^3^ International Maize and Wheat Improvement Center (CIMMYT), Texcoco, Mexico; ^4^ Anyang Academy of Agricultural Sciences, Anyang, China; ^5^ Institute of Crop Sciences, Chinese Academy of Agricultural Sciences, Beijing, China

**Keywords:** *Triticum aestivum* L., fusarium head blight resistance, *Fusarium verticillioides*, QTL mapping, genomic prediction

## Abstract

*Fusarium* head blight (FHB), is one of the destructive fugue diseases of wheat worldwide caused by the *Fusarium verticillioides* (*F.v*). In this study, a population consisting of 262 recombinant inbred lines (RILs) derived from Zhongmai 578 and Jimai 22 was used to map Quantitative Trait Locus (QTL) for FHB resistance, with the genotype data using the wheat 50 K single nucleotide polymorphism (SNP) array. The percentage of symptomatic spikelet (PSS) and the weighted average of PSS (PSSW) were collected for each RIL to represent their resistance to wheat head blight caused by *F.v*. In total, 22 QTL associated with FHB resistance were identified on chromosomes 1D, 2B, 3B, 4A, 5D, 7A, 7B, and 7D, respectively, from which 10 and 12 QTL were detected from PSS and PSSW respectively, explaining 3.82%–10.57% of the phenotypic variances using the inclusive composite interval mapping method. One novel QTL, *Qfhb. haust-4A.1*, was identified, explaining 10.56% of the phenotypic variation. One stable QTL, *Qfhb. haust-1D.1* was detected on chromosome 1D across multiple environments explaining 4.39%–5.70% of the phenotypic variation. Forty-seven candidate genes related to disease resistance were found in the interval of *Qfhb. haust-1D.1* and *Qfhb. haust-4A.1*. Genomic prediction accuracies were estimated from the five-fold cross-validation scheme ranging from 0.34 to 0.40 for PSS, and from 0.34 to 0.39 for PSSW in *in-vivo* inoculation treatment. This study provided new insight into the genetic analysis of resistance to wheat head blight caused by *F.v*, and genomic selection (GS) as a potential approach for improving the resistance of wheat head blight.

## Introduction

Wheat head blight, a disastrous disease found in temperate and subtropical areas around the world, results in severe yield losses, grain quality reduction, and even food safety crises (Buerstmayr et al., 2020). Its outbreaks have become more serious and more frequent in recent decades all over the world, especially in China, which has expanded greatly from the middle and lower reaches of the Yangtze River to the entire Yellow and Huai River Valleys region (Chen et al., 2017; Zhu et al., 2021). *Fusarium graminearum Schwabe* (*F.g*) and *Fusarium verticillioides* (*F.v*) are the two major fungal species, which can cause wheat head blight (Bai and Shaner, 1994; McMullen et al., 1997; O’Donnell et al., 1998). Extensive genetic research has been carried out on the resistance of wheat head blight caused by *F.g.* However, few research have been done on *F.v*-induced wheat head bligh*.* With the rapid extension of the special maize-wheat rotation and straw-returning farming method in the Yellow and Huai River Valleys region, the total amount of *F.v* in this area shows a dramatically increasing trend in recent years (Liu et al., 2019). Hence, further research on *F.v* is needed to obtain a better understanding and solution (Sun et al., 2015).

The complex resistance mechanisms have been divided into five types (Mesterházy et al., 1999) including evaluating the resistance to the initial infection (type 1), spread rate along the rachilla (type 2), mycotoxins accumulation (type 3), kernel damage rate (type 4), and host tolerance to the disease (type 5), respectively (Schroeder and Christensen, 1963; Miller et al., 1989; Mesterházy, 1995). The resistance of type 2 was usually inoculated and assessed on the live plant in the field by the single-floret inoculation (Mengist, 2013) and evaluated by the percentage of symptomatic spikelet (PSS) (Bai and Shang, 2004). However, all aforementioned studies were for *F.g-*induced FHB*,* therefore, more inoculation and assessment methods should be used to make the research on *F.v* more precise.

Currently, the main method of control for FHB is still chemical pesticides, which not only cause environmental pollution but also increase production costs. Therefore, it is very important to improve the effective resistance of wheat varieties to FHB (Ma et al., 2020). To date, seven resistance genes to FHB have been studied, including *Fhb1*, *Fhb2*, *Fhb3*, *Fhb4*, *Fhb5*, *Fhb6*, and *Fhb7* (Bai et al., 1999; Cuthbert et al., 2007; Qi et al., 2008; Xue et al., 2010, 2011; Cainong et al., 2015; Guo et al., 2015). Besides *Fhb1*, a new FHB resistance gene *Fhb7* was cloned recently from *Thinopyrum elongatum*, a distant wild relative of wheat (Wang et al., 2020). Using genetic mapping populations and association mapping, so far more than 500 FHB resistance-related QTL have been reported, distributed across all 21 chromosomes of wheat (Handa et al., 2008; Buerstmayr et al., 2009; Liu et al., 2009; Löffler et al., 2009; Steiner et al., 2017; Jia et al., 2018; Chen et al., 2021), among which most are derived from Asian sources including ‘Sumai 3’ and ‘Wangshuibai’, with the contribution from European and South American wheat varieties like Arina, Renan, Fundulea 201R, and Frontana (Gervais et al., 2003; Shen et al., 2003; Steiner et al., 2004; Buerstmayr and Buerstmayr, 2015). Nevertheless, seldom QTL was associated with wheat head blight caused by *F.v.* Moreover, coming from non-adapted backgrounds, the incorporation of such QTL into breeding programs have often resulted in the simultaneous transfer of other undesirable plant architect characteristics, low yield, and decreased seed quality, particularly in environments where these sources of resistance are not adapted (Buerstmayr et al., 2009; Liu et al., 2013; Prasanna et al., 2021). Therefore, it is highly desirable to identify, characterize and deploy local widely used sources of genetic resistance.

As a new technique to Marker Assisted Selection Breeding (MAS), especially for improving complex traits, genomic selection (GS), also known as genomic prediction (GP), offers significant prospects in wheat (Sandhu et al., 2021). GS predicts individuals’ genomic-estimated breeding values (GEBVs) by evaluating the effects of genome-wide markers (Xu et al., 2020). Therefore, GS captures a greater proportion of the genetic variation of the target trait than MAS (Cao et al., 2021) and it has been widely used in gain yield (Velazco et al., 2019), gain quality (Sehgal et al., 2020), and their relative traits (Tsai et al., 2020), and disease research in wheat (Herter et al., 2019; Muqaddasi et al., 2019, 2021). Medium to high prediction accuracies were reported in these studies, which suggested that GS is a potential genomic tool for improving complex traits.

*Fusarium* head blight caused by the *F.v* has not been selected as an important breeding trait in traditional wheat breeding programs, however, the research progress has not been reported. Thus, in this study, wheat head blight caused by *F.v* were conducted in a Recombinant Inbred Line (RIL) population crossing by ZM578 and JM22 including 262 lines, genotyped with a wheat 50 k SNP array, and phenotyped in four environments with *ex-vivo* or *in-vivo* inoculation treatments. The objectives of this study were to 1) evaluate the effectiveness of *ex-vivo* and *in-vivo* inoculation treatments in a RIL population screened for wheat head blight in multiple location trials; 2) detect QTL conferring type 2 FHB resistance caused by *F.v*, and identify major and stable QTL across inoculation treatments and environments by different evaluation methods; and 3) investigate the effectiveness of GS for improving *F.v* caused wheat head blight resistance.

## Materials and methods

### Plant materials

Zhongmai 578 (ZM578) and Jimai 22 (JM22), developed by the Chinese Academy of Agricultural Sciences and Shandong Academy of Agricultural Sciences, respectively, are two widely used varieties with wide adaptability and excellent agronomic traits. A recombinant inbred line (RIL) population consisting of 262 lines was derived from the cross between ZM578 and JM22, using the single seed descent (SSD) method. The genotype data of the F_5_ generation were used for further QTL mapping and GP analysis.

### Field trials

The RILs and their parents were planted in the experimental fields in Luoyang (34°32′N, 112°16′E, LY), Xinxiang (34°53′N, 113°23′E, XX), and Shangqiu (33°43′N, 114°49′E, SQ), Henan province in 2020–2021 cropping season. The meteorology records of that cropping season in each location were shown in Supplementary Table S1. In each location, a randomized block design with three replications was applied, and each plot had one 1 m row spaced by 20 cm between rows. Fifteen seeds were sown evenly per row. The field management followed the local practices.

### Phenotypic evaluation

The *F. v* (Shi et al., 2017) with strong pathogenicity, which was isolated from 123 strains of Henan province where this study was conducted, kindly provided by Prof. Hongxia Yuan from the college of plant protection of Henan Agricultural University, was used for inoculation. Two inoculation treatments were applied, i.e., inoculation on the *ex-vivo* panicles in the lab and *in-vivo* panicles in the field at the early flowering stage.

Single-floret Inoculation was applied at the early flowering stage as described by Mengist (2013). To generate the inoculum, *F. verticillioides* were grown in potato dextrose broth, and spore concentrations were adjusted after 7–10 days using a hemocytometer. Details on preparing conidia suspension were previously described (Xia et al., 2022). Conidia suspension (approximately 2×10^6^ spores per ml) of *F. verticillioides* was injected into the far-right floret of the fourth or fifth spikelet from the top of each spike (Duan et al., 2022), with a volume limited to 10 µl.

For the *ex-vivo* inoculation, three spikes of each line in RIL population and their parents were collected from each location in LY and SQ, and inoculation was applied at the laboratory. All inoculated spikes were placed in buckets with water and sealed with a black plastic bag on the top to provide a dark environment and high humid conditions favorable for wheat head blight infection. Wheat head blight severity was assessed 7 days after inoculation.

For *in-vivo* inoculation applied in the field, the infection and expansion conditions for each spike were controlled artificially to avoid the effect of the weather. To see whether other pathogens existed, the glume and grain have been cultured before and after inoculation, and the results demonstrated that there was no F. graminearum or other fungi that can cause ear disease. After which, three spikes of each RIL and their parents at the early following stage were selected and inoculated in the same way as the *ex-vivo* single-floret inoculation, and the spikes were covered with a plastic bag for moisturizing. The plastic bags were removed 72 h later, water was sprayed on the spikes for moisturizing for 18 days. The inoculated spikes were mist-irrigated twice per day, at 10 a.m. and 2 p.m., respectively. Wheat head blight severity assessments were evaluated 21 days after inoculation.

In the present study, the combination of location (LY, XX, SQ) and inoculation treatment (*ex-vivo* inoculation and *in-vivo* inoculation) was treated as one environment. In total, each RIL was evaluated in four environments. Meanwhile, PSS and PSSW were treated as two target traits.

### Wheat head blight severity assessment

Both PSS and PSSW were used to assess wheat head blight severity for both *ex-vivo* and *in-vivo* inoculation. The evaluation unit of PSS is the spikelet, whereas, that of PSSW is the kernel. The weighted factor depended on how many kernels were infected in the three kernels of each infected spikelet. The information of the number of the total spikelet (*N*_*TS*_), the spikelet with three kernels infected (*N*_*3k*_), the spikelet with two kernels infected (*N*_*2k*_), and the spikelet with only one kernel infected (*N*_*1k*_) was collected. The formulas for calculating PSS and PSSW are as follows:

$$PSS=\left(N_{3k}+N_{2k}+N_{1k}\right)/N_{TS}*100\%$$

$$PSSW=\left(N_{3k}+2/3*N_{2k}+1/3*N_{1k}\right)/\;N_{TS}*100\%$$

Where 1/3 referred one kernel was infected in the three kernels of each infected spikelet, 2/3 referred two kernels were infected in the three kernels of each infected spikelet, and one referred all three kernels were infected in each infected spikelet.

### Phenotypic data analysis

Pearson correlation analysis on PSS and PSSW of different environments was performed using IBM SPSS Statistics 22.0 (IBM, United States).

MEATA-R software (Alvarado et al., 2015) was used to analyze the multi-location trials using a mixed linear model to estimate the best linear unbiased estimation (BLUE) and heritability (*H* ^*2*^). BLUE value of genotype in and across environments for further analysis. The mixed linear model was applied as follows:

$$Y_{ijk}=µ+G_{i}+E_{j}+{GE}_{ij}+R_{k}E_{j}+e_{ijk}$$

where *Y*_*ijk*_ is the target trait, *µ* is the overall mean, *G*_*i*_, *E*_*j*_, and *GE*_*ij*_ are the effects of the *i*th genotype, *j*th environment, and *i*th genotype by *j*th environment interaction, respectively. *R*_*k*_ *E*_*j*_ is the effect of the *k*th replication within the *j*th environment. *E*_*ijk*_ is the residual effect of the *i*th genotype, *j*th environment, and *k*th replication. Genotype is considered as a fixed effect, whereas all other terms are declared as random effects.

Broad-sense heritability (*H* ^*2*^) of each environment was calculated using

$$H^{2}=\frac{V_{G}}{V_{G}+\frac{V_{G\times E}}{n}+\frac{V_{e}}{nr}}$$

where *V*_*G*_ is the genotypic variance, *V*_*G×E*_ is the variance component of the genotype-by-environment interaction, *V*_*e*_ represents the residual variance, *n* is the number of environments, and *r* is the number of replicates in each environment.

### Genotyping and linkage map construction

Genomic DNA for SNP assays was extracted from young leaf tissues by the CTAB method (Clarke, 2009). The 262 RILs and two parents were genotyped using 50 K SNP assay (Sun et al., 2020). Markers with non-polymorphism, missing data greater than 10%, and minor allele frequency less than 0.30 were excluded from the further linkage mapping analysis using TASSEL v5.0 (Bradbury et al., 2017). The “BIN” function in QTL IciMapping 4.1 software (Meng et al., 2015) was used to remove the redundant markers. Linkage groups were constructed with the “MAP” function in QTL IciMapping 4.1, and the chromosome information of the linkage maps was distinguished by using the physical position of SNPs on the Chinese spring reference genome (IWGSC) RefSeq v1.0. (The International Wheat Genome Sequencing Conosrtium, 2018).

### QTL mapping

QTL mapping was conducted using the “BIP” function in QTL IciMapping 4.1 (Meng et al., 2015), and the algorithm of inclusive composite interval mapping was selected. The walking step for QTL detection was set as 0.1 cM, and the LOD score threshold was set as 2.5, which was used to declare the putative QTL. The additive effect (Add) and phenotypic variation explained (PVE) of each QTL were estimated. Each QTL detected from each individual and the combined environment was defined as an individual QTL, whereas the QTLs located in the same physical position was defined as a unique QTL. The QTL with a PVE value greater than 10% was defined as major QTL, and the QTL detected in at least three environments was defined as a stable QTL.

### Identification and *in silico* expression analysis of candidate genes

The sequence information of the left and right markers of the stable QTL were used to blast to the Chinese spring reference genome on the website EnsemblPlants (http://plants.ensembl.org/Multi/Tools/Blast) and to identify the physical interval on the reference genome. Within the physical interval of each stable QTL, candidate genes were identified based on the information on the Wheat Gmap website (https://www.wheatgmap.org/tools/gene/information/), and the functions of the candidate gene involved in disease resistance were annotated as well. *In silico* expression analysis of candidate genes was to see if there are any reported expression for these genes against fusarium in wheat, which was done on Wheat Expression Browser website (http://www.wheat-expression.com) (Borrill et al., 2016).

### Genomic prediction

Genomic prediction analysis was implemented in the *rrBLUP* package (Endelman, 2011) to estimate the prediction accuracy of PSS and PSSW within each environment and in combinedENV. All the 1,507 SNPs used in the genetic map were applied for genomic prediction analysis (Zhao et al., 2012). Details of the implementation of *rrBLUP* were described earlier (Endelman, 2011). A five-fold cross-validation scheme with 100 replications was used to generate the training and validation sets, and to assess the prediction accuracy. The average value of the correlations between the phenotype and the genomic estimated breeding values was defined as genomic prediction accuracy (*r*_*MG*_).

## Results

### Phenotypic symptoms of wheat head blight caused by *F.v*

**FIGURE 1:**
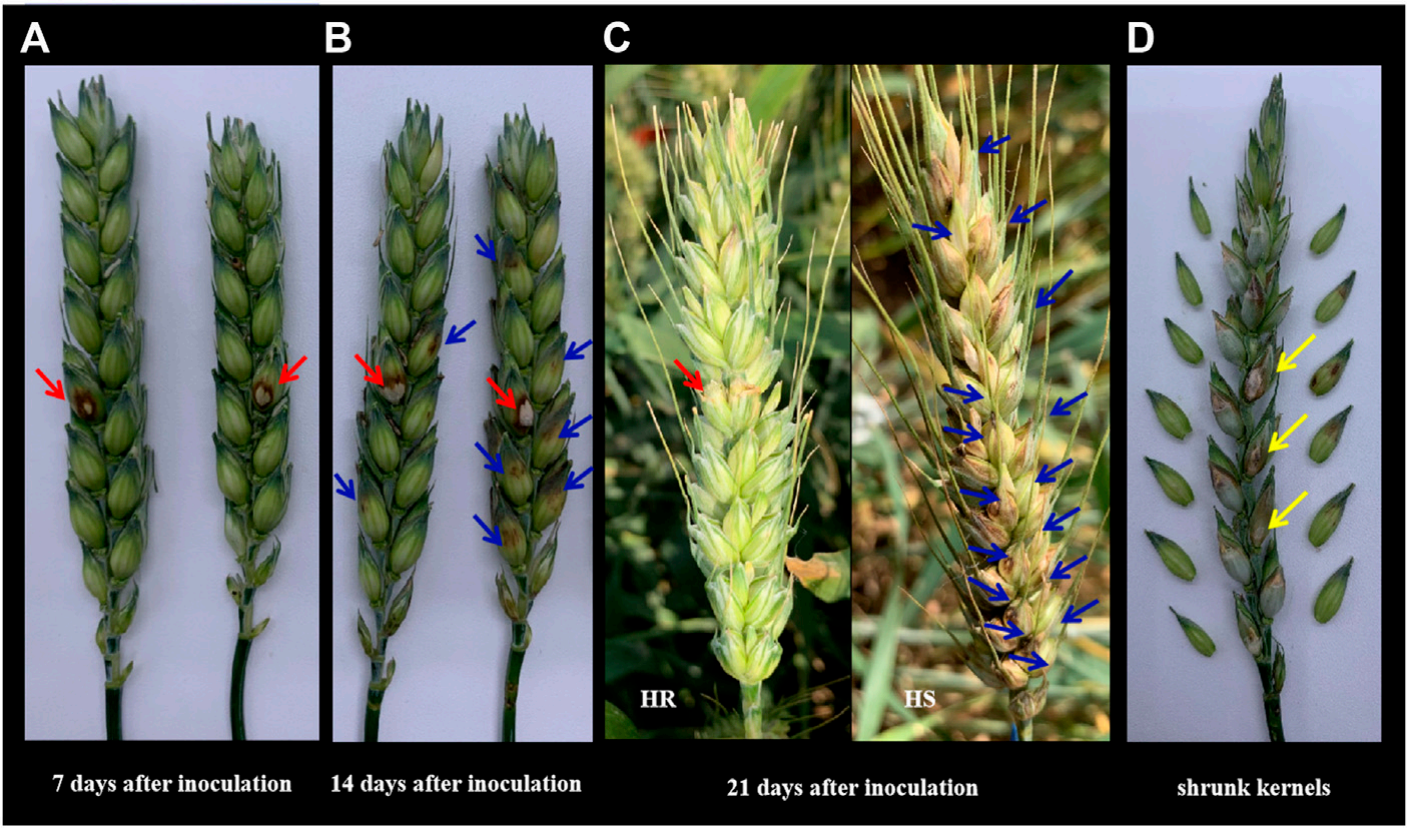
The phenotypic symptoms to *F.v* on the spikelet. **(A)** The response symptom on the inoculation site of two different lines in the population. Photos were taken 7 days after single floret inoculation. Red arrows indicated the inoculation site. **(B)** The expansion symptom of 14 days after inoculation on different lines in the population. Red arrows showed the inoculation site while the blue ones showed the expansion severity in the above and below directions. **(C)** The expansion symptom of 21 days after inoculation on the lines with the highest resistance (HR) and the lines with the highest susceptible (HS) in this population in the fields. Red arrows showed the inoculation site while the blue ones showed the expansion severity in the above and below directions. **(D)** Shrunk kernels on inoculation and other infected sites.

The *Fv.* Inoculated kernels had obvious brown spots (red arrows shown in Figures 1A–C), which was the main symptom of the glume. The infection area spread and expanded around the inoculation site (blue arrows shown in Figures 1B,C), leading to the shrunk grains (yellow arrows shown in Figure 1D). The disease development process in 7 days, 14 days, and 21 day after inoculation was shown in Figures 1A–C. The symptom of the highest resistance (HR) line and highest susceptible (HS) line in this population were shown in Figure 1C.

### Phenotypic evaluation

Detailed temperature and rainfall records of each location could be found in Supplementary Table S1, which indicated that minor differences were shown in the temperature conditions, whereas the total quantity of rainfall was more in LY and SQ almost twice that in XX. However, the artificial micro-environment, like covering the plastic bag on each spike to ensure the infection condition and mist-irrigation twice per day to ensure the high moisture for expansion, ensured that the experiment would not be affected by the external environment.

**FIGURE 2:**
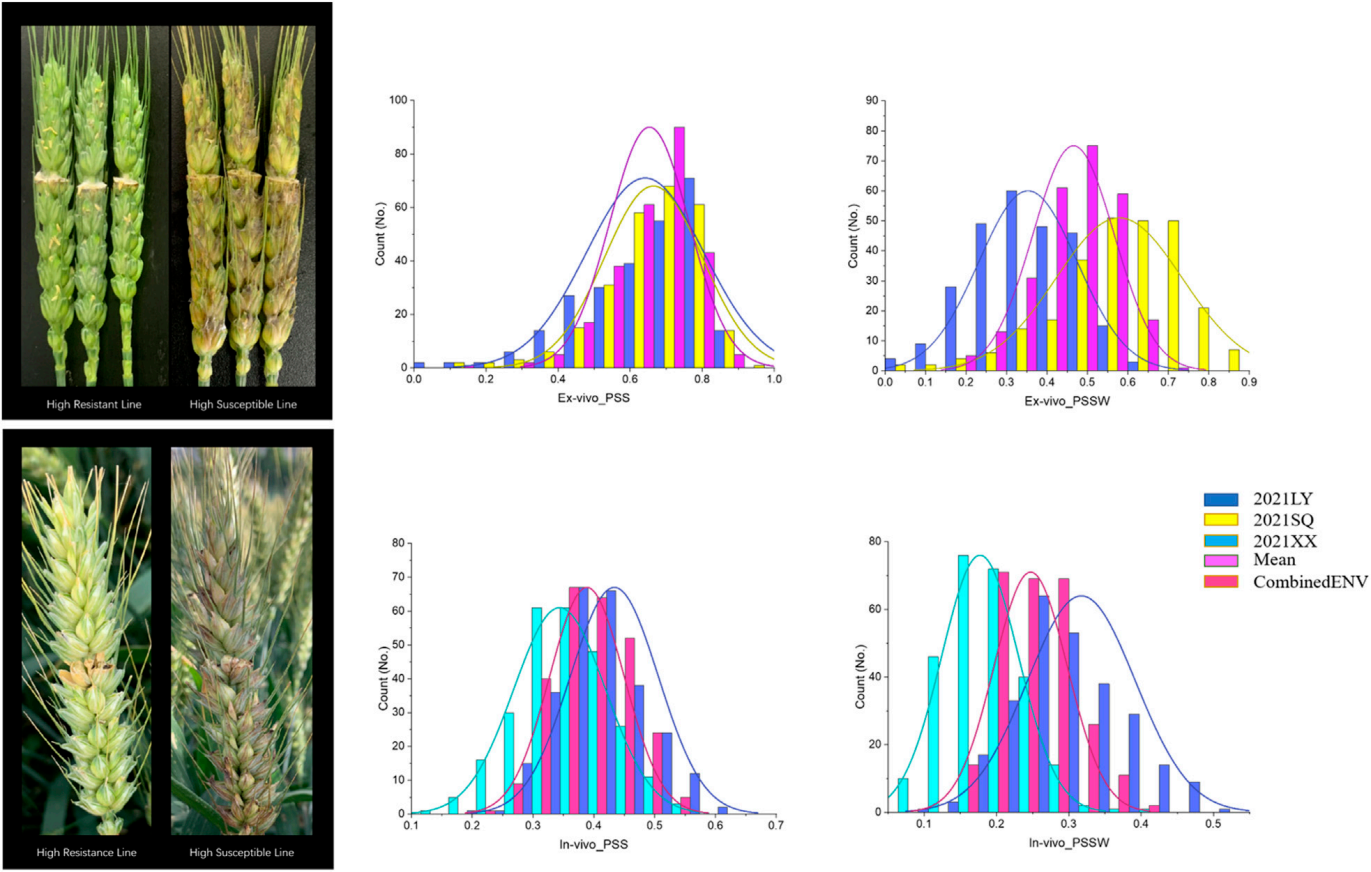
The highest resistant and susceptible lines and the distribution for PSS and PSSW of in the ZM 578/JM 22 population in each location.

For the *ex-vivo* single-floret inoculation treatment on the spike at the early flowering stage in the LY location, PSS of JM22 was extremely significantly lower than that of ZM578. In the SQ location, the same trend was observed. In both LY and SQ locations, broad variations were observed in PSS, ranging from 0.00 to 0.98 with an overall mean of 0.63 in the LY location, and from 0.11 to 1.00 with an overall mean of 0.66 in the SQ location (Table 1; Figure 2).

**TABLE 1:** Phenotypic variation, heritability (H^*2*^) and ANOVA analysis of PSS and PSSW for ZM578, JM22, and the derived RILs in *Ex-vivo* and *In-vivo* inoculation treatment in different locations.

Inoculation site	Treatment	Trait	Location	Parents		Population			H^2^	ANOVA		
				ZM578	JM22	Min	Max	Mean		V_G_	V_G×E_	V_e_
Panicles	*Ex-vivo*	PSS	LY	0.45 ± 0.00	0.04 ± 0.003**	0.00	0.98	0.63 ± 0.01	—			
			SQ	0.64 ± 0.05	0.54 ± 0.03	0.11	1.00	0.66 ± 0.009	—			
		PSSW	LY	0.16 ± 0.006	0.02 ± 0.006**	0.00	0.69	0.35 ± 0.01	—			
			SQ	0.49 ± 0.06	0.30 ± 0.01*	0.04	1.00	0.58 ± 0.009	—			
	*In-vivo*	PSS	LY	0.56 ± 0.04	0.51 ± 0.05	0.10	0.84	0.44 ± 0.003	0.40	2.042^***^	299.831^***^	0.551
			XX	0.38 ± 0.04	0.30 ± 0.06	0.00	0.82	0.34 ± 0.003	0.50			
			CombinedENV	0.43	0.39	0.24	0.45	0.34 ± 0.002	0.52			
		PSSW	LY	0.44 ± 0.04	0.42 ± 0.04	0.03	0.84	0.32 ± 0.003	0.46	1.862^***^	880.283^***^	0.854
			XX	0.14 ± 0.02	0.15 ± 0.04	0.00	0.76	0.17 ± 0.002	0.53			
			CombinedENV	0.28	0.26	0.11	0.30	0.17 ± 0.002	0.47			

*, **, *** indicate significant level at *p* < 0.05, 0.01, 0.001, respectively.

The same trend was observed for PSSW as that for PSS. JM22 has lower PSSW than that of ZM578 both in LY and SQ locations, with a highly significant and significant difference, respectively. A wide variation was observed in PSSW, ranging from 0.00 to 0.69 with an overall mean of 0.35 in the LY location, and ranging from 0.04 to 1.00 with an overall mean of 0.58 in the SQ location (Table 1; Figure 2).

In the *in-vivo* single-floret inoculation treatment, PSS values of JM22 were also lower than those of ZM578 in all the locations. The values were 0.56s ± 0.04, 0.38 ± 0.04, 0.43 for ZM578, 0.51 ± 0.05, 0.30 ± 0.06, and 0.39 for JM 22 in LY, XX, and CombinedENV, respectively. The mean PSS values of all the RILs were 0.44.0.34, and 0.34 in LY, XX, and CombinedENV, respectively. The PSS values of all the RILs ranged from 0.10 to 0.84, from 0.00 to 0.82, and from 0.24 to 0.54 in LY, XX, and CombinedENV, respectively. The heritabilities of PSS *in-vivo* single-floret inoculation treatment were moderate ranging from 0.40 to 0.52 in individual location and CombinedENV.

ANOVA indicated that both PSS and PSSW were significantly influenced by genotype and genotype by environment interaction effects, with the genotype by environment interaction contributing the most (Table 1). A similar trend was observed for PSSW (Table 1).

### Pearson correlation analysis on PSS and PSSW of different environments

The distribution and the results of Pearson correlations between PSS and PSSW, as well as those for the same trait between different environments, were shown in Table 2. In the *ex-vivo* inoculation experiments at the early flowering stage, very low correlations between locations were observed for both PSS and PSSW, as well as between PSS and PSSW from different locations. Within the same location, correlations between PSS and PSSW were very high and greater than 0.90.

**TABLE 2:** Correlation coefficients of PSS and PSSW across different inoculation treatments and locations.

Correlation		Treatment	*Ex-vivo* inoculation				*In-vivo* inoculation					
		Trait	PSS		PSSW		PSS			PSSW		
Treatment	Trait	Location	LY	SQ	LY	SQ	LY	XX	CombinedENV	LY	XX	CombinedENV
*Ex-vivo* inoculation	PSS	LY										
		SQ	0.063									
	PSSW	LY	0.902**	0.065								
		SQ	0.055	0.975**	0.075							
*In-vivo* inoculation	PSS	LY	0.064	0.137*	0.085	0.147*						
		XX	0.02	0.158*	0.027	0.160**	0.347**					
		CombinedENV	0.046	0.180**	0.06	0.187**	0.732**	0.892**				
	PSSW	LY	0.091	0.058	0.099	0.064	0.925**	0.330**	0.684**			
		XX	0.081	0.191**	0.091	0.210**	0.343**	0.881**	0.804**	0.320**		
		CombinedENV	0.107	0.150*	0.117	0.165**	0.792**	0.733**	0.913**	0.826**	0.798**	

*, ** indicate significant level at *P* < 0.05 and 0.01, respectively

In the *in-vivo* single-floret inoculation experiments, the correlation coefficient for the same trait between locations was 0.347 and 0.3 for PSS and PSSW, respectively. For both PSS and PSSW, the correlation coefficients between individual location and CombinedENV were relatively high, i.e., greater than 0.73. While the correlation coefficients for the same trait between different locations were not high, which was between 0.35 and 0.30 for PSS and PSSW, respectively. The correlation coefficients between PSS and PSSW in individual location ranged from 0.88 to 0.93. The correlation coefficients between PSS and PSSW ranged from 0.68 to 0.80 between the individual locations and CombinedENV, while the correlation coefficient between PSS and PSSW was 0.913 between two CombinedENV.

### Linkage map constructed

Linkage map of this population constructed by selected 1,507 markers, all of which were assigned to 39 linkage groups (Table 3). The total length of the genetic map was 2,413.84 cM, with an average distance of 1.60 cM between markers. The distance between the two linked markers ranged from 0.36 to 6.01 cM in the A genome, from 0.26 to 3.18 cM in the B genome, and from 0.41 to 8.91 cM in the D genome. The number of SNP differed greatly among genomes and chromosomes. Compared to A and B genomes, the D genome had fewer SNPs. Genome A, B, and D had 568, 545, and 394 SNPs, respectively. The maximum number of SNP was found on chromosome 7B having 155 SNPs, and the lowest number of SNP was mapped on chromosome 6A having 11 SNPs.

**TABLE 3:** Genetic linkage map information.

Chr^a^	LG^b^	No^c^.of SNP^d^	Genetic length/cM	Mean density of markers/cM	Chr	LG	No.of SNP	Genetic length/cM	Mean density of markers/cM	Chr	LG	No.of SNP	Genetic length/cM	Mean density of markers/cM
1A	1	115	195.24	1.70	1B	2	42	10.77	0.26	1D	3	5	2.06	0.41
2A	5	3	18.03	6.01	2B	8	132	142.30	1.08		4	89	164.50	1.85
	6	39	34.24	0.88	3B	13	52	42.08	0.81	2D	9	10	5.98	0.60
	7	27	35.24	1.31		14	19	60.48	3.18		10	45	79.95	1.78
3A	12	100	123.33	1.23	4B	19	12	11.47	0.96		11	10	39.62	3.96
4A	16	27	61.73	2.29	5B	23	79	66.71	0.84	3D	15	7	12.24	1.75
	17	18	10.79	0.60		24	54	95.28	1.76	4D	20	46	81.25	1.77
	18	28	53.00	1.89	7B	34	13	36.58	2.81	5D	25	15	22.27	1.48
5A	21	7	4.24	0.61		35	59	53.29	0.90		26	23	204.97	8.91
	22	57	180.70	3.17		36	16	10.35	0.65	6D	29	13	9.86	0.76
6A	27	7	11.43	1.63		37	67	56.30	0.84		30	33	91.22	2.76
	28	4	1.44	0.36							31	5	2.35	0.47
7A	32	133	171.94	1.29						7D	38	8	34.78	4.35
	33	3	3.16	1.05							39	85	172.70	2.03
**A genome**	14	568	904.50	1.59	**B genome**	11	545	585.61	1.07	**D genome**	14	394	923.73	2.34

^a^ Chromosome.

^b^ Linkage group.

^c^ Number of SNP.

^d^ Single Nucleotide Polymorphism.

### QTL mapping of wheat head blight resistance caused by *F.v*

In total, twenty-two individual QTLs were detected for wheat head blight resistance to *F.v* (Supplementary Table S2). In the *ex-vivo* inoculation experiments, three individual QTLs were detected (Supplementary Table S2), including one individual QTL for PSS from the LY location, and two individual QTLs for PSSW in both LY and SQ locations. In the *in-vivo* inoculation environments, 12 individual QTLs were detected including six individual QTLs for PSS in LY and XX locations, and six individual QTLs for PSSW in LY and XX locations. The rest of seven individual QTLs were detected in CombinedENV, in which 3 individual QTLs for PSS and four individual QTLs for PSSW. Finally, 11 unique QTLs have been detected (Supplementary Table S2).

The distribution of the detected QTLs on each chromosome was shown in Supplementary Table S2. For PSS, 10 individual QTLs were detected and mapped on chromosomes 1D (4), 4A (2), 3B (1), 7B (1), 5D (1), and 7D (1) (Supplementary Table S2). On chromosome 1D, four individual QTLs were detected in individual locations and combinedENV, which were considered as a stable QTL across environments. Two individual QTLs were detected on chromosome 4A, while only one individual QTL was detected on chromosome 3B, 7B, 5D, and 7D, respectively.

For PSSW, 12 individual QTLs were detected and mapped on chromosomes 1D (3), 7A (3), 7D (2), 4A (2), 2B (1), and 5D (1) (Supplementary Table S2). On chromosome1D and 7A, three individual QTLs, one unique QTL was detected in individual locations and combinedENV on each chromosome, which was considered as a stable QTL across environments. Two individual QTLs were detected on chromosomes 7D and 4A respectively, while only one QTL was detected on chromosomes 2B and 5D, respectively.

**FIGURE 3:**
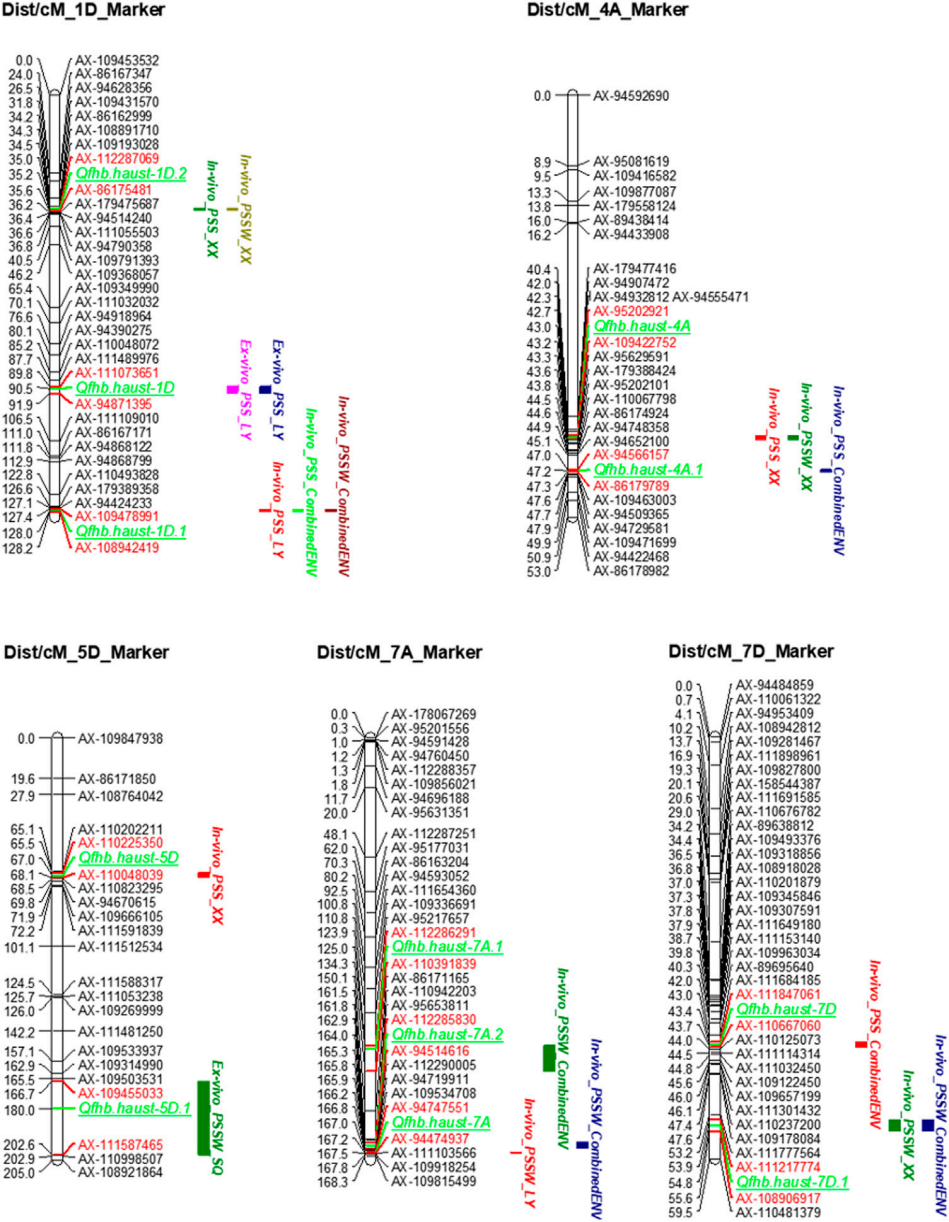
Quantitative trait loci (QTL) for *Fusarium* head blight resistance identified in the ZM 578/JM 22 population. QTLs were detected in more than two environments. The positions of marker loci are shown on the right of the linkage groups and centiMorgan (cM) distances between loci are shown along the left. The environments where the QTLs were detected are shown in different colors on the right of the linkage groups. The flanking markers of each QTL are highlighted in red color.

The QTLs have been detected in more than two environments were shown in Table 4 and Figure 3. The environments where the QTLs were detected were distinguished in different colors on the right of the linkage groups in Figure 3. The stable QTL on chromosome 1D was detected across all three individual locations and CombinedENV(Table 4; Figure 3). The LOD values of these seven QTLs ranged from 2.90 to 4.91, and their PVE values ranged from 3.82% to 5.81% (Table 4). However, the genetic and corresponding physical positions of these seven QTLs were different and divided into three genomic regions, namely *Qfhb. haust-1D*, *Qfhb. haust-1D.1*, and *Qfhb. haust-1D.2*. Moreover, *Qfhb. haust-1D.1* was also repeatedly detected for both traits of PSS and PSSW.

**TABLE 4:** Quantitative trait loci (QTL) detected in more than two environments for *Fusarium* head blight resistance mapped in the ZM 578/JM 22 population assessed by PSS and PSSW.

QTL	Trait, Environment^a^	QTL					
		Chr^b^	Physical interval (Mb)	Flanking markers	LOD^c^	PVE^d^ (%)	Add^e^
*Qfhb.haust-1D*	*Ex-vivo*, PSS, LY	1D	340.55–356.15	AX-111073651—AX-94871,395	3.03	5.12	-0.06
	*Ex-vivo*, PSSW, LY	1D	340.55–356.15	AX-111073651—AX-94871,395	3.16	5.38	-0.04
***Qfhb.haust-1D.1***	*In-vivo*, PSS, LY	1D	434.03–436.14	AX-109478991—AX-108942419	3	4.39	-0.01
	*In-vivo*, PSS, CombinedENV	1D	434.03–436.14	AX-109478991—AX-108942419	3.15	5.1	-0.01
	*In-vivo*, PSSW, CombinedENV	1D	434.03–436.14	AX-109478991—AX-108942419	4.91	5.7	-0.01
*Qfhb.haust-1D.2*	*In-vivo*, PSS, XX	1D	8.62–10.72	AX-112287069—AX-86175481	4.72	5.81	-0.01
	*In-vivo*, PSSW, XX	1D	8.62–10.72	AX-112287069—AX-86175481	2.9	3.82	-0.01
*Qfhb.haust-4A*	*In-vivo*, PSS, XX	4A	712.73—	AX-95202921—AX-109422752	5.38	6.69	-0.01
	*In-vivo*, PSSW, XX	4A	712.73—	AX-95202921—AX-109422752	6.06	8.51	-0.01
***Qfhb.haust-4A.1***	*In-vivo*, PSS, CombinedENV	4A	714.85–717.97	AX-94566157—AX-86179789	6.36	10.57	-0.01
*Qfhb.haust-7D*	*In-vivo*, PSS, CombinedENV	7D	393.88–395.19	AX-111847061—AX-110667060	3.51	5.68	-0.01
*Qfhb.haust-7D.1*	*In-vivo*, PSSW, XX	7D	511.07–512.70	AX-111217774—AX-108906917	3.12	4.38	-0.01
	*In-vivo*, PSSW, CombinedENV	7D	511.07–512.70	AX-111217774—AX-108906917	3.3	3.93	-0.01
*Qfhb.haust-5D*	*In-vivo*, PSS, XX	5D	459.06–465.00	AX-110225350— AX-110048039	3.26	4.1	-0.01
*Qfhb.haust-5D.1*	*Ex-vivo*, PSSW, SQ	5D	549.03–560.39	AX-109455033—AX-111587465	2.69	4.73	0.04
*Qfhb.haust-7A*	*In-vivo*, PSSW, LY	7A	676.08–677.70	AX-94747551— AX-94474937	2.95	4.74	0.01
*Qfhb.haust-7A.1*	*In-vivo*, PSSW, CombinedENV	7A	534.33–608.88	AX-112286291— AX-110391839	5.18	6.02	-0.01
*Qfhb.haust-7A.2*	*In-vivo*, PSSW, CombinedENV	7A	671.47–674.27	AX-112285830—AX-94514616	3.43	4.06	0.01

^a^ Environment = Location×inoculation treatment (*ex-vivo*/*in-vivo* inoculation).

^b^ Chromosome.

^c^ logarithm of the odds.

^d^ phenotypic variation explained.

^e^ Add, estimated additive effects of QTL, at the current scanning position. Positive and negative values indicate that the resistance alleles are inherited from ZM578 and JM, 22, respectively.

The main QTL on chromosome 4A was detected in one individual location and CombinedENV. The LOD values of these three QTLs ranged from 5.38 to 6.36, and their PVE values ranged from 6.69% to 10.56% (Table 4). However, the genetic and corresponding physical positions of these three QTLs were different and divided into two genomic regions, namely *Qfhb. haust-4A* and *Qfhb. haust-4A.1*, respectively. Moreover, *Qfhb. haust-4A.1* was the one with the highest PVE among all repeated QTL we detected. Therefore, it could be considered a major QTL. As a result, the consequence gene screening should be done on *Qfhb. haust-1D.1* and *Qfhb. haust-4A.1*.

The unique QTL on chromosome 7D was detected in one individual location and CombinedENV. The LOD values of this unique QTL ranged from 3.12 to 3.51, and their PVE values ranged from 3.93% to 5.68% (Table 4). One unique QTL on chromosome 7A was detected in one individual environment and CombinedENV as well. The LOD values of this unique QTL ranged from 2.95 to 5.18, and their PVE values ranged from 4.06% to 6.02% (Table 4). However, the favorable allele of this loci was shown to arise from different parents. The rest six unique QTLs for wheat head blight resistance caused by *F.v* were detected in less than three environments, explaining 3.82%–6.98% of the phenotypic variance, with the LOD value ranging from 2.66 to 4.80.

### *In silico* expression analysis for putative candidate genes associated with wheat head blight resistance

**FIGURE 4:**
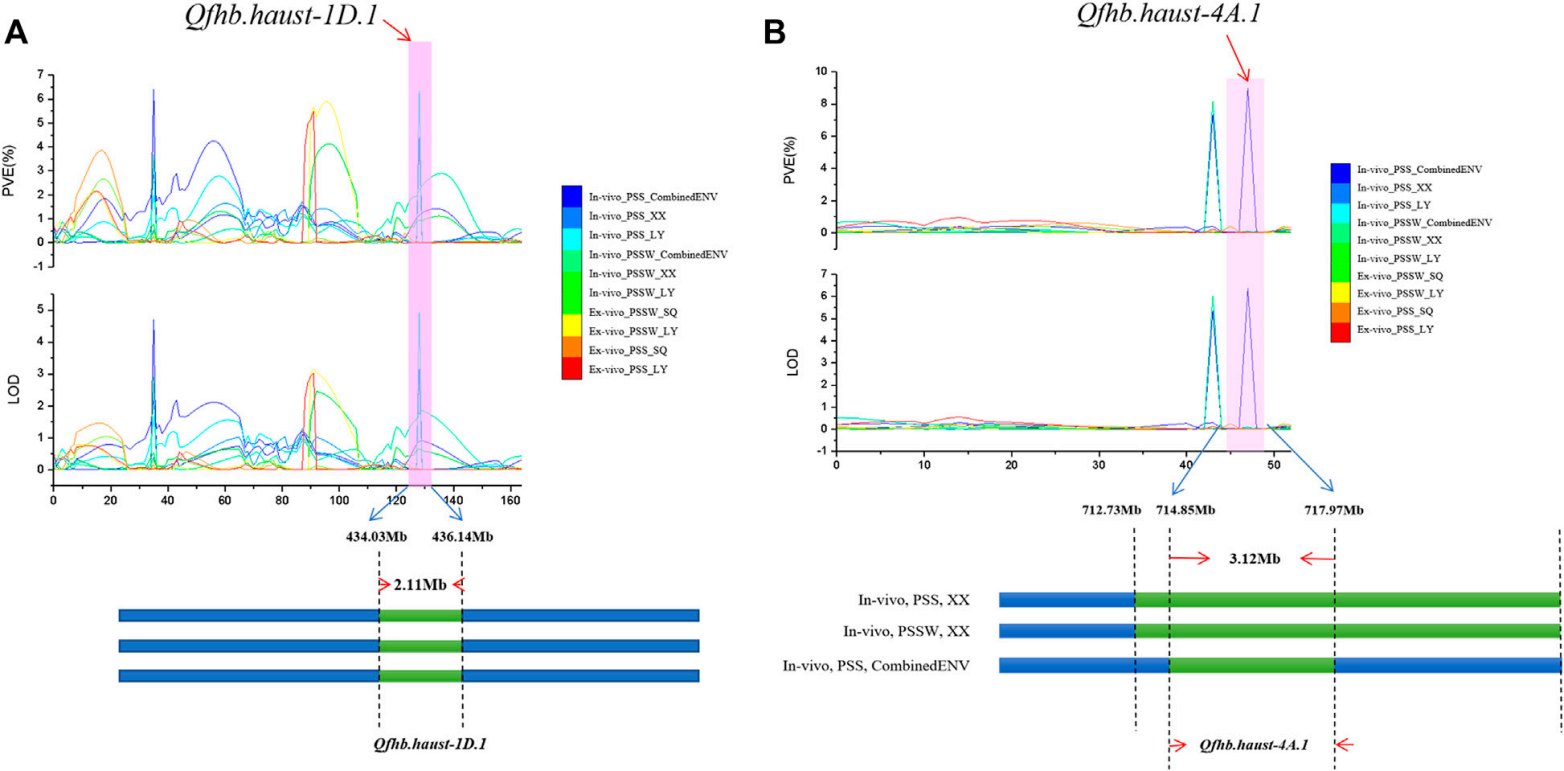
The LOD and PVE values of the whole linkage group 1D and 4A, the physical location and length of *Qfhb. haust-1D.1* **(A)** and *Qfhb. haust-4A.1* **(B)**.

**FIGURE 5:**
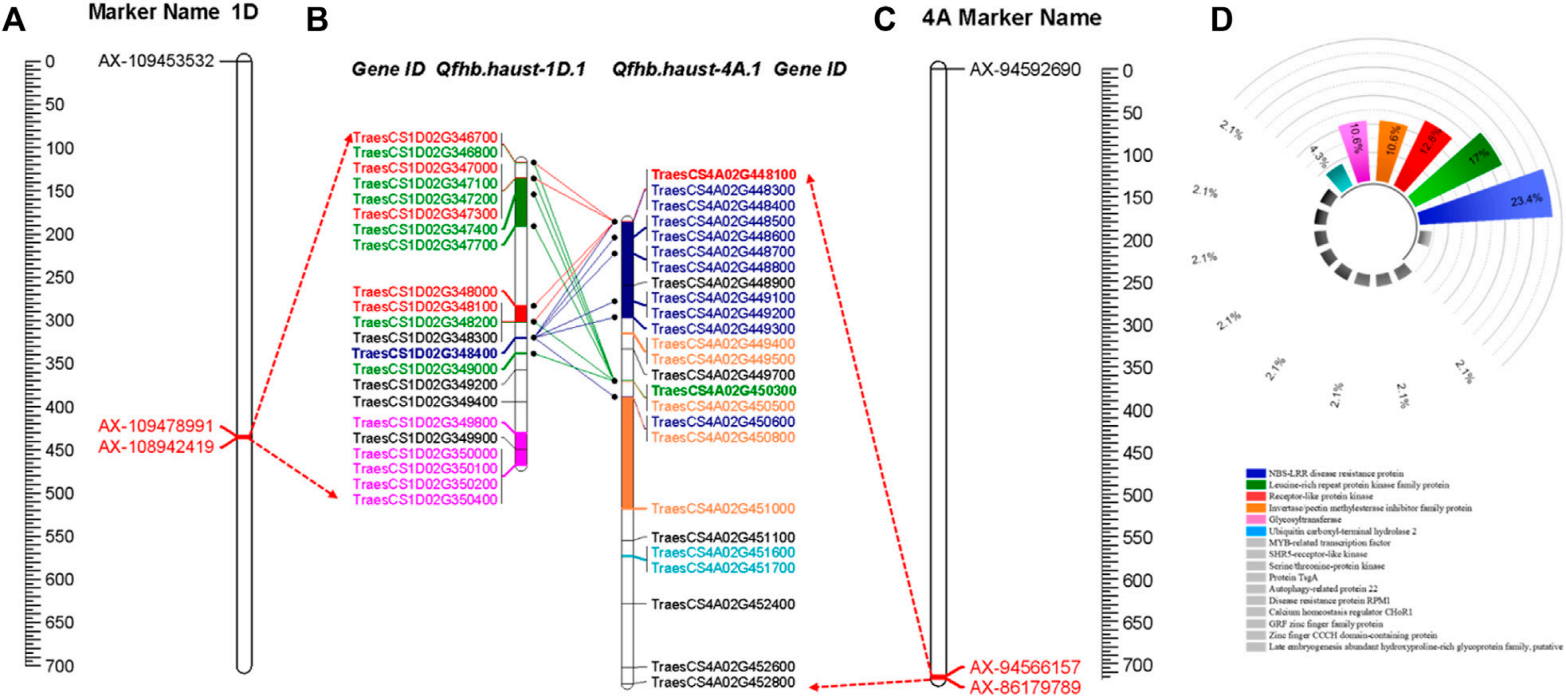
The distribution of the putative candidate genes associated with wheat head blight resistance located in the intervals of *Qfhb. haust-1D.1* and *Qfhb. haust-4A.1*. **(A)** The location of *Qfhb. haust-1D.1* on chromosome 1D. **(B)** Putative candidate genes with the same function were shown in the same color, and the gene with the same function but located on different chromosomes were connected by the lines between the chromosomes. **(C)** The location of *Qfhb. haust-4A.1* on chromosome 4A. **(D)** The proportion of each function among these 47 putative candidate genes and the colors were the same color with the gene ID of **(B)**.

Based on the candidate gene analysis, the physical position of *Qfhb. haust-1D.1* was mapped on chromosome 1D in the interval of 434.03 Mb–436.14 Mb with a distance of 2.11 Mb (Figure 4A). *Qfhb. haust-4A.1* was mapped on chromosome 4A in the interval of 714.855 Mb–717.97 Mb with a distance of 3.12 Mb (Figure 4B). In total, 192 candidate genes existed in these two intervals, and 95 genes were left after removing ones with low confidence (LC). Based on the annotation information of these candidate genes, 47 putative candidate genes were selected with the potential of their functions being involved in response to disease. Among the 47 genes, 22 candidate genes were in the interval of *Qfhb. haust-1D.1*, and the rest 25 candidate genes were in the interval of *Qfhb. haust-4A.1* (Figure 5). Sixteen potential functions were covered (Figure 5), from which NBS-LRR disease resistance protein, Leucine-rich repeat protein kinase family protein, and receptor-like protein kinase were the largest three proportions accounting for 23.4%, 17.0%, and 12.8%, respectively.

**FIGURE 6:**
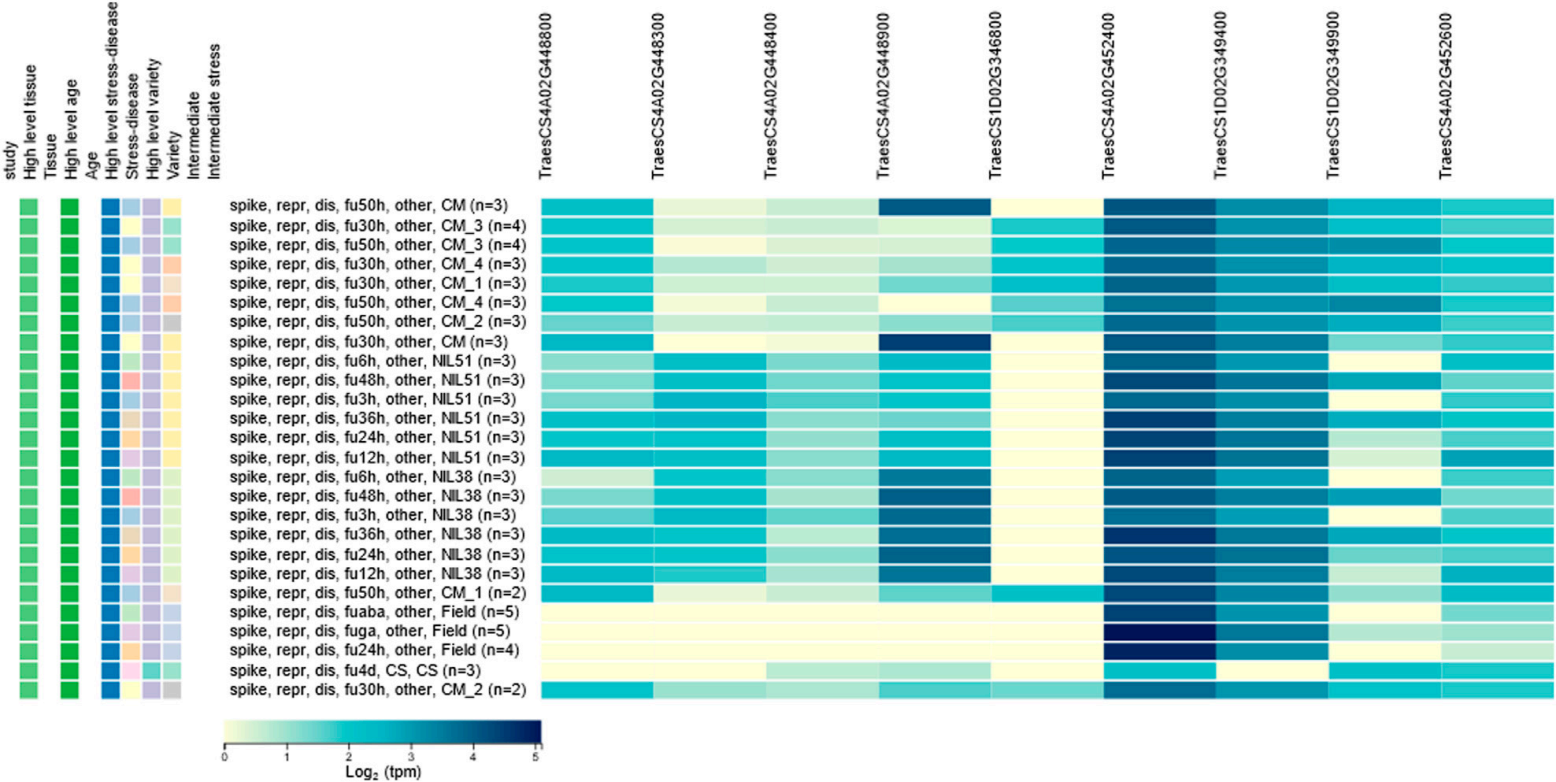
*In silico* expression analysis for candidate genes. Nine genes were reported expression against fusarium several hours after inoculation.

The 47 candidate genes were used to do the *in silico* expression analysis. The RNA-seq data of these genes are represented using a heatmap (Figure 6). Based on the *in silico* analysis of gene expression data and gene annotations, nine candidate genes, including *TraesCS1D02G346800, TraesCS1D02G349400,* and *TraesCS1D02G349900* underlying on *Qfhb. haust-1D.1,* and *TraesCS4A02G448800, TraesCS4A02G448300, TraesCS4A02G448400, TraesCS4A02G448900, TraesCS4A02G452400,* and *TraesCS4A02G452600* underlying on *Qfhb. haust-4A.1,* were reported expression against fusarium several hours after inoculation*.*

### Genomic prediction accuracies estimated from the five-fold cross-validation schemes

**FIGURE 7:**
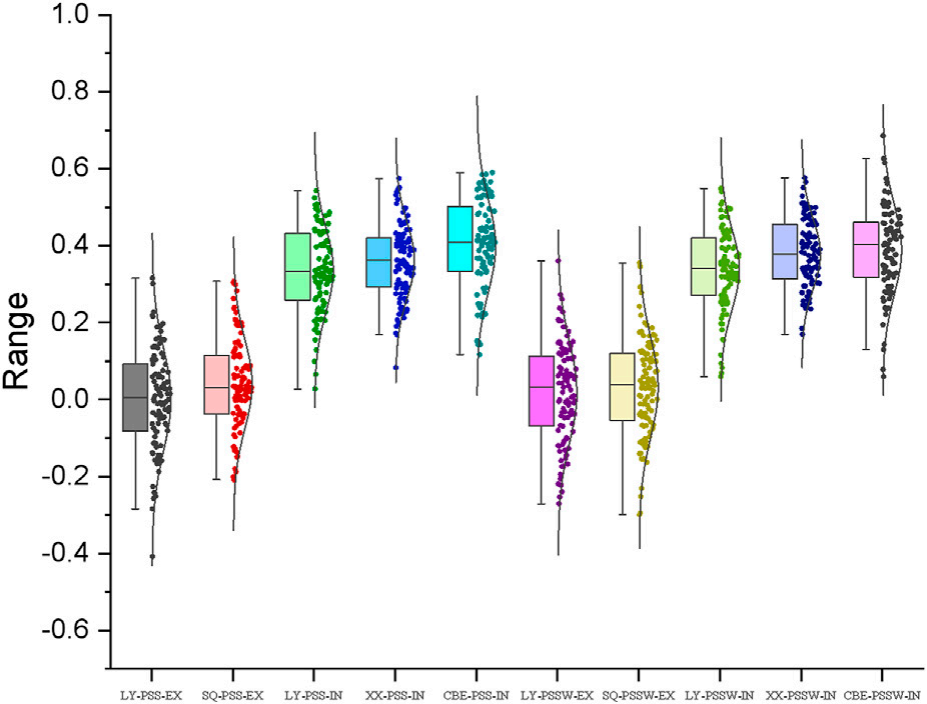
Genomic prediction (GP) accuracies for FHB resistance estimated from the five-fold cross-validation schemes with the genome-wide markers by *ex-vivo* (EX) and *in-vivo* (IN) inoculation treatments in Luoyang (LY), Xinxiang (XX), and Shangqiu (SQ) locations assessed by PSS and PSSW.

The prediction accuracies of wheat head blight resistance estimated from the five-fold cross-validation schemes were shown in Figure 7. In the *ex-vivo* inoculation experiments, the prediction accuracies were close to 0 for both traits in all the environments. In the *in-vivo* inoculation experiments, the average prediction accuracies ranged from 0.34 to 0.40 for PSS and 0.34 to 0.39 for PSSW. The accuracies estimated from the combinedENV were higher than those estimated from individual environment for both traits, which was consistent with the heritability result.

## Discussion

### Comparison of the *ex-vivo* and *in-vivo* inoculation treatments

The quantitative nature of this complex trait brings inherent difficulties in the phenotyping of wheat head blight resistance due to the confounding effects of the volatile environment and the various genetic background. To achieve more reliable phenotypic data, an artificial inoculation (single-floret inoculation) was used in the present study to keep high humidity during the whole infection period and repeat the experiment in different locations. In addition, *in-vivo* inoculation in the field and *ex-vivo* inoculation were also conducted in the laboratory. While the *in-vivo* inoculation was a classic approach to utilize, the temperature and moisture in the lab for inoculation on *ex-vivo* panicles could be easier to control. It could potentially save a lot of labor if it works.

It is expected that *Ex-vivo* inoculation should produce more stable and repeatable results. However, our result suggests it is not the case. The speculative reasons might include: 1) A large number of materials were placed together and covered in the bucket, and as a result, the moisture may be too high and not even; and 2) Mutual infection between adjacent spikelets made the results not reliable enough. The data quality of the *ex-vivo* inoculation experiment in the present study was not sufficient, and it is unlikely to be a suitable way to assess the severity of wheat head blight caused by *F.v*.

### Comparison of the major and/or stable QTL identified

Identifying and verifying available FHB resistance genes of different pathogenic is essential for resistance improvement in wheat breeding (Carpenter et al., 2020). In this study, two QTL were identified associated with wheat head blight caused by *F.v*, compared with the previously reported QTL associated with *F.g*-induced FHB, because there was no report on *F.v*-induced wheat head blight previously.

To date, not many QTL associated with FHB was reported on chromosome 1D. IWGSC_CSS_1DS_scaff_1879930_3352 was detected by Marcio (2015) in a 273 breeding panel, which was located on chromosome 1D at 19.04 cM, associating with DON. Another one QTL associated with wheat head blight resistance was found that explained 7.2% of phenotypic variation, located between 229.7 and 291.7 Mb on chromosome 1D (Goddard R et al., 2021). It indicated that *Qfhb. haust-1D.1* found in this research with the genomic interval of 434.03–436.14 Mb (127.38–128.22 cM) on chromosome 1D (Table 4) is a novel QTL.

Several QTLs for FHB resistance were reported on chromosome 4A previously (Yang et al., 2005; Liu et al., 2009; McCartney et al., 2016). Steed et al. (2005) mapped a QTL near *Xgwm165* at 51.12 Mb, using a set of 21 substitution lines of *Triticum* macha in a ‘Hobbit Sib’ background, resistance to initial infection. Kollers et al. (2013) using 358 recent European winter wheat varieties plus 14 spring wheat varieties mapped a QTL between *cfa2256* at 81.7 Mb and *Xcfd71* at 146.7 Mb with the resistance to spread of infection. Agnes et al. (2014) mapped a QTL near *wPt-0804* around 18.0 Mb in Frontana, explaining 14.6% effect of the phenotypic variation. *Qfhb. nc-4A.1a* and *Qfhb. nc-4A.2a* were mapped in intervals 24.3–49.4 Mb and 67.0–114.5 Mb, explaining 11.6%–23.3% and 17.4%–20.0% effect of the phenotypic variation, respectively (Petersen et al., 2017). Furthermore, another one QTL from Zhengmai 9,023 was mapped in the interval 132.9 and 310.3 Mb, explaining 5.08% effect of the phenotypic variation (Zhang et al., 2021). And Zhu et al. (2021) mapped a QTL from Zhongmai 895, located between 12.6 and 12.9 Mb on chromosome 4A, explaining 3.2%–8.0% effect of the phenotypic variation. *Qfhb. haust-4A.1* mapped in the current study was located between 714.85 and 717.97 Mb, which is probably a novel QTL associated with *F.v*-induced wheat head blight, indicating that the resistance QTL for different pathogens are more likely different.

Of the 47 genes with predicted function, nine genes showed responses to fusarium inoculation based on the *in silico* expression analysis (Figure 6). Three genes belonged to NBS-LRR disease resistance protein family which are the majority of disease resistance genes in plants (Youssef et al., 2004). One gene, TraesCS1D02G346800 belonged to a leucine-rich repeat receptor-like protein family, which also has the LRR domains. LRR domains have long been implicated in plant disease resistance (Jones and Jones, 1997), and also have been found in other plants’ resistant studies (Liu et al., 2021). Furthermore, gene *TraesCS4A02G448900* from chromosome 4A, encoding disease resistance protein RPM1 (Boyes et al., 1998), could trigger a defense system including the hypersensitive response that restricts the pathogen growth. Other four genes were found associated with Calcium homeostasis regulator CHoR, autophagy-related protein 22, Late embryogenesis abundant hydroxyproline-rich glycoprotein family and MYB-related transcription factor, which were believed to be involved in the plant defense processes (Wu et al., 2001). Further work is needed for fine mapping, Nevertheless, the candidate genes and *in silico* expression analysis conducted in this study have provided the basis for fine mapping *Qfhb. haust-1D.1* and *Qfhb. haust-4A.1*.

### Genomic prediction for wheat head blight caused by *F.v*

Previously published studies and the present study revealed that wheat head blight resistance is controlled by multiple minor QTL with small effects and is highly influenced by the genetic background of the population studies (Zhang et al., 2021), which implied that MAS for improving wheat head blight resistance may not be very effective.

Genomic Prediction is effective and powerful for the improvement of complex traits in wheat. Some of the previous studies focused on complex diseases, such as Tan Spotcaused by Pyrenophora tritici-repentis (Ptr) (Muqaddasi et al., 2019) and Septoria tritici blotch (STB) caused by Zymoseptoria tritici (Muqaddasi et al., 2021) whereas most of the studies focused on the grain-related traits (Velazco et al., 2019; Tsai et al., 2020; Sandhu et al., 2021). Based on their studies, the prediction accuracies of grain-related traits varied from 0.8 to 0.95, whereas that of both two diseases ranged from 0.4 to 0.5 which showed the same in this study, indicating that the prediction accuracy was closely connected with the heritability of each trait. In the present study, the prediction accuracy of *F.v*-induced wheat head blight was around 0.4, which was closely correlated with heritability, which indicated that the results were reasonable and that genomic prediction could be used for *F.v*-induced wheat head blight. GS is a promising tool for improving *F.v*-induced wheat head blight resistance, nevertheless, in practical breeding, improving the GP accuracy is a key target in future studies.

## Data Availability

The original contributions presented in the study are included in the article/Supplementary Material, further inquiries can be directed to the corresponding authors.
