# Supplementary material for: QTL mapping and genomic prediction of resistance to wheat head blight caused by Fusarium verticillioides
Source: Front Genet. 2022 Oct 24;13:1039841. doi: 10.3389/fgene.2022.1039841 (PMC9638129; doi:10.3389/fgene.2022.1039841)
Supplement: Supplementary file 1 [file DataSheet1.docx]

**Supplementary TABLE 1 | The weather record of each experiment location**

| Month | Location | T_max_ (℃)**^a^** | T_min_ (℃) **^b^** | Average of high temperature (℃) | Average of low temperature (℃) | Total Rainfall (mm) |
| --- | --- | --- | --- | --- | --- | --- |
| May | LY | 36 | 10 | 29 | 16 | 8.06 |
|  | XX | 38 | 10 | 29 | 16 | 0.91 |
|  | SQ | 35 | 9 | 27 | 15 | 28.51 |
| June | LY | 38 | 17 | 34 | 22 | 8.30 |
|  | XX | 39 | 16 | 34 | 22 | 38.84 |
|  | SQ | 39 | 15 | 34 | 22 | 72.61 |
| July | LY | 39 | 19 | 32 | 23 | 392.63 |
|  | XX | 37 | 22 | 33 | 23 | 24.89 |
|  | SQ | 37 | 19 | 32 | 23 | 372.08 |
| August | LY | 33 | 17 | 29 | 21 | 100.12 |
|  | XX | 34 | 17 | 30 | 22 | 120.40 |
|  | SQ | 34 | 17 | 29 | 21 | 178.24 |
| September | LY | 33 | 14 | 26 | 18 | 98.60 |
|  | XX | 33 | 13 | 27 | 18 | 184.41 |
|  | SQ | 35 | 14 | 28 | 18 | 53.65 |
| Octobor | LY | 28 | 5 | 19 | 10 | 19.64 |
|  | XX | 29 | 2 | 20 | 10 | 28.81 |
|  | SQ | 33 | 1 | 21 | 10 | 15.70 |

a: The highest temperature of this month.

b: The lowest temperature of this month.

| **Supplementary TABLE 2 \|** Quantitative trait loci (QTL) mapping for Fusarium head blight resistance in the ZM 578/JM 22 population | | | | | | | | |
| --- | --- | --- | --- | --- | --- | --- | --- | --- |
| Traits | Environment^a^ | QTL | | | | | | |
|  |  | Chr.^b^ | Position | Interval (cM) | Flanking markers | LOD^d^ | PVE^e^ (%) | Add^f^ |
| PSS | Ex-vivo, LY | 1D | 91 | 89.77—91.87 | AX-111073651—AX-94871395 | 3.03 | 5.12 | -0.06 |
|  | In-vivo, LY | 1D | 128 | 127.38—128.22 | AX-109478991—AX-108942419 | 3 | 4.39 | -0.01 |
|  | In-vivo, LY | 3B | 31 | 30.16—31.68 | AX-108785780—AX-95233993 | 3.14 | 4.67 | 0.01 |
|  | In-vivo, LY | 7B | 14 | 13.99—14.96 | AX-86167574—AX-94907220 | 2.66 | 3.82 | 0.01 |
|  | In-vivo, XX | 1D | 35 | 34.98—35.57 | AX-112287069—AX-86175481 | 4.72 | 5.81 | -0.01 |
|  | In-vivo, XX | 4A | 43 | 42.75—43.22 | AX-95202921—AX-109422752 | 5.38 | 6.69 | -0.01 |
|  | In-vivo, XX | 5D | 66 | 65.47—68.13 | AX-110225350— AX-110048039 | 3.26 | 4.1 | -0.01 |
|  | In-vivo, CombinedENV | 1D | 128 | 127.38—128.22 | AX-109478991—AX-108942419 | 3.15 | 5.1 | -0.01 |
|  | In-vivo, CombinedENV | 4A | 47 | 46.98—47.32 | AX-94566157—AX-86179789 | 6.36 | 10.57 | -0.01 |
|  | In-vivo, CombinedENV | 7D | 43 | 42.99—43.67 | AX-111847061—AX-110667060 | 3.51 | 5.68 | -0.01 |
| PSSW | Ex-vivo, LY | 1D | 91 | 89.77—91.87 | AX-111073651—AX-94871395 | 3.16 | 5.38 | -0.04 |
|  | Ex-vivo, SQ | 5D | 200 | 166.73—202.59 | AX-109455033—AX-111587465 | 2.69 | 4.73 | 0.04 |
|  | In-vivo, LY | 7A | 167 | 166.78—167.23 | AX-94747551— AX-94474937 | 2.95 | 4.74 | 0.01 |
|  | In-vivo, XX | 1D | 35 | 34.98—35.57 | AX-112287069—AX-86175481 | 2.9 | 3.82 | -0.01 |
|  | In-vivo, XX | 2B | 98 | 97.90—98.06 | AX-111057916—AX-94937912 | 4.8 | 6.47 | 0.01 |
|  | In-vivo, XX | 4A | 24 | 1.64—27.98 | AX-109882531—AX-94715337 | 3.64 | 6.98 | 0.01 |
|  | In-vivo, XX | 4A | 43 | 42.75—43.22 | AX-95202921—AX-109422752 | 6.06 | 8.51 | -0.01 |
|  | In-vivo, XX | 7D | 55 | 53.89—55.63 | AX-111217774—AX-108906917 | 3.12 | 4.38 | -0.01 |
|  | In-vivo, CombinedENV | 1D | 128 | 127.38—128.22 | AX-109478991—AX-108942419 | 4.91 | 5.7 | -0.01 |
|  | In-vivo, CombinedENV | 7A | 124 | 123.9—134.33 | AX-112286291— AX-110391839 | 5.18 | 6.02 | -0.01 |
|  | In-vivo, CombinedENV | 7A | 164 | 162.94—165.25 | AX-112285830—AX-94514616 | 3.43 | 4.06 | 0.01 |
|  | In-vivo, CombinedENV | 7D | 55 | 53.89—55.63 | AX-111217774—AX-108906917 | 3.3 | 3.93 | -0.01 |
| a:Environment = Location×inoculation treatment (ex-vivo/ in-vivo inoculation) | | | | | | | | |
| b: Chromosome | | | | | | | | |
